# Anxiety, depression, and memory loss in Chagas disease: a puzzle far beyond neuroinflammation to be unpicked and solved^*^

**DOI:** 10.1590/0074-02760220287

**Published:** 2023-04-03

**Authors:** Joseli Lannes-Vieira, Glaucia Vilar-Pereira, Leda Castaño Barrios, Andrea Alice Silva

**Affiliations:** 1 Fundação Oswaldo Cruz-Fiocruz, Instituto Oswaldo Cruz, Laboratório de Biologia das Interações, Rio de Janeiro, RJ, Brasil; 2 Universidade Federal Fluminense, Faculdade de Medicina, Departamento de Patologia, Laboratório Multidisciplinar de Apoio à Pesquisa em Nefrologia e Ciências Médicas, Niterói, RJ, Brasil

**Keywords:** Chagas disease, neurological disorders, behavioural changes, neurocognitive disorders, anxiety, depression, memory loss, preclinical model, benznidazole, immunotherapy

## Abstract

Mental disorders such as anxiety, depression, and memory loss have been described in patients with chronic Chagas disease (CD), a neglected tropical disease caused by the protozoan parasite *Trypanosoma cruzi*. Social, psychological, and biological stressors may take part in these processes. There is a consensus on the recognition of an acute nervous form of CD. In chronic CD patients, a neurological form is associated with immunosuppression and neurobehavioural changes as sequelae of stroke. The chronic nervous form of CD has been refuted, based on the absence of histopathological lesions and neuroinflammation; however, computed tomography shows brain atrophy. Overall, in preclinical models of chronic *T. cruzi* infection in the absence of neuroinflammation, behavioural disorders such as anxiety and depression, and memory loss are related to brain atrophy, parasite persistence, oxidative stress, and cytokine production in the central nervous system. Interferon-gamma (IFNγ)-bearing microglial cells are colocalised with astrocytes carrying *T. cruzi* amastigote forms. *In vitro* studies suggest that IFNγ fuels astrocyte infection by *T. cruzi* and implicate IFNγ-stimulated infected astrocytes as sources of TNF and nitric oxide, which may also contribute to parasite persistence in the brain tissue and promote behavioural and neurocognitive changes. Preclinical trials in chronically infected mice targeting the TNF pathway or the parasite opened paths for therapeutic approaches with a beneficial impact on depression and memory loss. Despite the path taken, replicating aspects of the chronic CD and testing therapeutic schemes in preclinical models, these findings may get lost in translation as the chronic nervous form of CD does not fulfil biomedical model requirements, as the presence of neuroinflammation, to be recognised. It is hoped that brain atrophy and behavioural and neurocognitive changes are sufficient traits to bring the attention of researchers to study the biological and molecular basis of the central nervous system commitment in chronic CD.

Mental health disorders: a challenge to be faced in this century

Behavioural and neurocognitive changes are mental health disorders diagnosable by clinical signs and symptoms. In a simplified way, these changes reflect suffering about time: anxiety is the suffering for what has not yet been lived; depression is the suffering with what has been lived or is being lived, and the memory loss or mnemonic alteration reflects the loss of what was experienced. The improvement in living conditions, due to technological development and investments in public health (sanitation, vaccinations) and access to medicaments (such as antibiotics), led to a reduction in the number of deaths from infectious diseases, allowing aging and an increase in the frequency of deaths from chronic degenerative diseases, as cardiovascular and neurodegenerative disorders.^1^ ^,^ ^2^ In 2019, around 15% (1-in 7-people) of the world’s population exhibit one or more mental health or substance use disorders, among which the most frequent are anxiety and depression. In Brazil, 17% of the population suffers with some mental health disorder.^3^

Behavioural and neurocognitive changes, common findings in aging, degenerative disorders, and chronic infections, may be linked by sequential relationships or common biological and molecular mechanisms. More recently, the gut microbiota has been proposed as a determinant of the mental health.^4^ Also, a bulk of data suggest the pivotal participation of previous immune experience triggered by infectious, progressive immunosenescence, dysregulation of the immune response, and chronic inflammation in behavioural and neurocognitive disorders.^1^ ^,^ ^5^ ^,^ ^6^ In this context, the study of Chagas disease (CD), a chronic parasitic illness, may help us to understand the contribution of a long-term invader/host crosstalk in mental health performance. Chronic CD shows a spectrum of clinical forms (indeterminate, cardiac, gastrointestinal) and systemic immunological alterations. Moreover, a variety of behavioural and neurocognitive changes occur in parallel with the presence of the parasite in the central nervous system (CNS), devoid of neuroinflammation. Here, we will review these points, the proposed cellular and molecular mechanisms underpinning these processes, and discuss promising perspectives to ameliorate the quality of life of chronic CD patients.

Chagas disease: a general overview

American trypanosomiasis or CD is a neglected tropical disease that affects 6 to 7 million people, while an estimated 75 million are at risk of infection, according to the WHO.^7^ Further, CD is part of the panel of neglected diseases that affects 1 in 7 inhabitants of the planet.^8^ This illness was described by Carlos Chagas more than 110 years ago. He also discovered its etiological agent, the hemoflagellate protozoan parasite *Trypanosoma cruzi*, and its biological cycle, which involves invertebrate hosts and vectors, the triatomine insect family, and mammalian vertebrate hosts, such as man.^9^ In vertebrate hosts, this parasite, which potentially infects all cell types from macrophages to all cell types of the CNS, replicates as amastigote forms in the cell cytoplasm; these intracellular forms differentiate into trypomastigote forms, which disrupt the host cell, infecting new cells in a tissue, or spreading through the bloodstream or other fluids to other tissues (Fig. 1A). CD is endemic in America, currently, however, due to human migration in search of better living conditions is considered globalised. Despite the population decrease of vectors, due to control and surveillance programs,^10^ there are still reports of a high rate of home invasion by CD vectors in endemic regions.^11^ The most important form of CD transmission is still the vector-born (either via the lesion resulting from the bite, or via the ocular or oral mucosa), blood-borne, and congenital.^10^ Presently, the oral form of CD transmission is the most mentioned in Brazil, occurring via ingestion of food, mainly fruit juices (açai, sugar cane, guava, cashew) contaminated with debris or excreta of the vector. Control initiatives by PAHO and the Health Ministries of the affected countries have significantly reduced vectorial transmission in Latin America.^10^ However, transfusion transmission and organ transplantation are still of epidemiological importance, due to the inexistence or failure of blood test control. Unfortunately, congenital transmission causes about 14,000 births of infected children per year and became a central target of control initiatives (such as the “Cuida Chagas Project”), involving Brazil, Bolivia, Colombia, and Paraguay and supported by international consortia sponsored by Brazilian Health Ministry and UNITAID.^12^

The infection by *T. cruzi* has two phases, acute and chronic. The acute phase, usually asymptomatic or with nonspecific symptoms (fever, lymph node infarction, oedema) presents patent parasitism in the blood (parasitaemia) and tissues, such as the CNS, heart, and peripheral muscles.^10^ ^,^ ^13^ After four to eight weeks, the immune response is established (innate and adaptive, with pivotal participation of macrophages, dendritic cells, antibodies, and T cells), leading to control of parasitaemia and tissue parasitism, biomarkers of onset of the chronic phase.^13^ In this phase, the presence of parasites in blood and tissues is rare and intermittent, being, however, detected by conventional microscopy (rare intracellular forms), immunohistochemistry, and PCR for detection of *T. cruzi* DNA.^10^

The progression of infection and the clinical outcome forms are dependent on a complex host/invader interplay that involves environmental and genetic factors of both the host and the infectious agent.^14^ In CD more recent studies are contradictory, ruling out or pointing to the possibility of a genetic predisposition of patients to the development of clinical forms of the chronic phase of CD,^15^ ^,^ ^16^ ^,^ ^17^ ^,^ ^18^ reinforcing that more robust studies, with a greater number of patients and ethnical and geographic diversities, are needed to draw more solid conclusions. Some hypotheses have been proposed to explain tissue damage and progression to organ dysfunction. Tissue injury may be directly induced by parasite persistence and/or indirectly by parasite-specific immune response or recognition of self-released antigens, contributing to dysregulated immune response and systemic inflammatory profile. Though, the pathophysiological factors underpinning the outcome of the host/parasite interaction are not entirely understood. There is a bulk of evidence that parasite persistence in tissues associated with ill-adapted homeostatic mechanisms, such as oxidative/anti-oxidative and pro-inflammatory/anti-inflammatory processes, are critical for the pathogenesis and progression of organ dysfunction in CD.^18^ ^,^ ^19^

Lack of prophylactic and therapeutic vaccines^20^ and effective treatment,^21^ especially for patients with the chronic form of the disease, remain challenges to be faced aiming to modify the natural history of CD. Another current challenge is the identification of clinical and laboratory biomarkers that indicate risk, and prognosis or may allow follow-up of therapeutic protocols. Most carriers of *T. cruzi* infection (60-70%) remain without symptoms and clinical signs, in the indeterminate form (IND). Depending on the studied group, clinical evolution from IND to the determinate forms ranges of 1.85-7% per year.^22^ The pathology is mainly characterised by the cardiac form (CARD), in 20-30% of patients, with progressive cardiomyopathy associated with myocarditis, fibrosis, electrical changes, and cardiac dysfunction. Characteristic electrocardiographic findings such as atrial fibrillation, flutter, and atrioventricular block are frequently associated with stroke in under-forty-year-old *T. cruzi*-infected patients.^23^ About 10% of the infected individuals develop the gastrointestinal form that can result in megacolon and/or megaoesophagus, which are often associated with the CARD, constituting the chronic cardio-digestive form.^22^ ^,^ ^23^ The existence of the chronic nervous form of CD is controversial.^24^ The lack of recognition of a chronic nervous form of CD may contribute to the neglect a substantial group of patients, hampering appropriate medical care and attention. Independent of formal acceptance of clinical classification, attention is demanded by chronic CD patients suffering from behavioural and neurocognitive disorders.^25^ Here, we are going to overview some aspects of this question, show our recent findings using preclinical models and critically discuss some challenges to be faced to overcome a long-time pointless contention, however relevant to dealing with the mental health issues of afflicted patients.

Nervous form of Chagas disease: consensus and overlooked points

Since Carlos Chagas first described the main clinical aspects of the acute phase of American trypanosomiasis, he proposed the existence of the acute nervous form of this disease, based on cases of fatal outcomes preceded by neurological changes and convulsion in children.^26^ Histological studies revealed meningoencephalitis, amastigote, and trypomastigote forms in neuroglia cells (Fig. 1B), supporting the life cycle of *T. cruzi* in the CNS, inflammatory nodules in the white matter of the cortex, and low-grade perivascular mononuclear infiltration.^27^ Importantly, parenchymal cells and neurons were morphologically preserved.^27^ These data were corroborated by studies showing that meningoencephalitis, mainly associated with myocarditis, can be fatal for children; however, if timely diagnosed and etiologically treated, the survival rate is increased.^24^ CNS commitment in acute infection in adults and the elderly remains an overllooked issue and deserves attention. Nevertheless, the acute nervous form of CD in children is a consensus and, more, a concern considering the number of cases of congenital transmission,^7^ the demand for diagnosis, adequate treatment, and follow-up of infected children to avoid CNS commitment and premature death.^12^ ^,^ ^28^

**Fig. 1:**
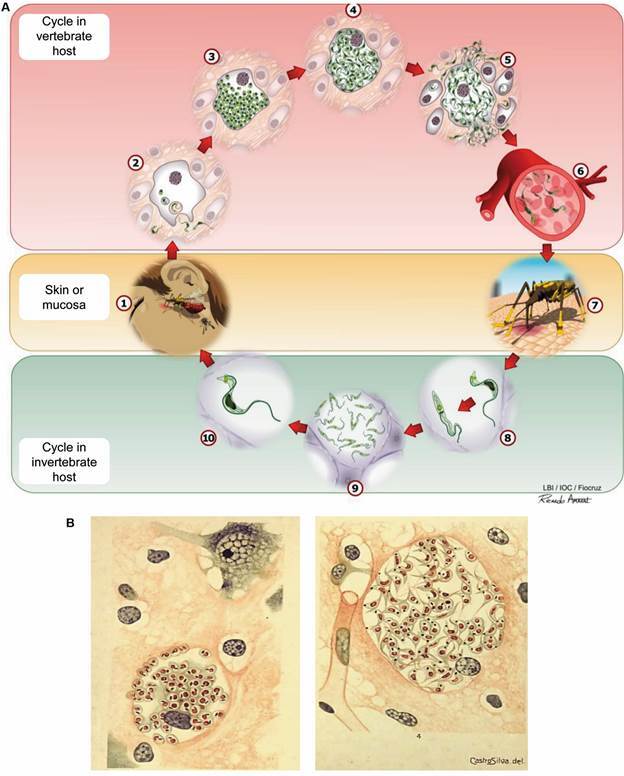
the cycle of life of *Trypanosoma cruzi*. (A) *T. cruzi* is naturally transmitted by the Hemiptera insect (kissing bug). During the blood meal, the insect deposits excreta (feces and urine) containing the infective forms (metacyclic trypomastigotes) of the parasite on the skin of the vertebrate host (1). Then, the metacyclic trypomastigote forms reach the underlying tissue and the host’s bloodstream through the insect bite, by spreading to injured areas, or through the mucous membranes (ocular, oral). In mammalian hosts, trypomastigotes invade local cells (2) and differentiate into amastigotes, which are intracellular and multiplicative forms of the parasite (3). A new differentiation occurs to infective trypomastigote forms (4) causing the rupture of the cell and release of the parasites (5). These trypomastigote forms can invade new cells, stay in the blood (6) or colonise other tissues. Hemiptera are infected when they ingest trypomastigotes during their blood meal (7). In the invertebrate host, ingested trypomastigotes evolve into epimastigotes (8), which multiply intensely along the midgut of the insect (9) and, when reaching the rectal ampulla, differentiate into metacyclic trypomastigotes (10), which are infective forms for the mammalian host (Collective creation of LBI/IOC; illustration commissioned to Ricardo Amaral). (B) Histological images of the central nervous tissue of an acute case of chagasic encephalitis showing the cycle of *T. cruzi* parasite in the brain tissue. (left) A neuroglial cell enlarged by numerous amastigote forms of *T. cruzi*. A neuron can be seen in the upper right part of the figure. (right) Neuroglial cell distended by a few amastigotes and numerous trypomastigote forms of *T. cruzi*. The nucleus of the parasitised cell is observable in the lower right of the figure. Reproduced from Vianna^27^ with the kind permission of the editor of the Memórias do Instituto Oswaldo Cruz.

Another consensus is the recognition of the reactivation of CD in the chronic phase in immunosuppressive conditions, as in human immunodeficiency virus (HIV) co-infection,^29^ transplanted or neoplasia-treated patients.^30^ ^,^ ^31^ This condition was reproduced in chronically *T. cruzi*-infected mice submitted to immunosuppressive conditions when brain infection was reactivated associated with increased expression of cell adhesion molecules by endothelial cells and neuroinflammation.^32^ In CD patients, neurological alterations such as headache, mental confusion, and convulsion are the main clinical signs of these conditions.^33^ Trypomastigote forms are detected in blood and cerebrospinal fluid, and computed tomography showed brain oedema and tumour-like lesion in the corpus callosum.^29^ Histologically, common findings are haemorrhagic necrotic nodules, tumour-like mass enriched in parasites, moderate focal and scattered inflammatory infiltrates, and parasite-bearing reactive macrophages.^29^ ^,^ ^33^ ^,^ ^34^ ^,^ ^35^ Crucially, if timely diagnosed the drug of choice to treat the reactivated infection in the CNS in immunosuppressed patients is benznidazole, which is accessible to the brain tissue,^36^ effective in controlling parasitism and parasitaemia, and in improving clinical outcomes and patient life expectancy.^29^ ^,^ ^35^

Chagas proposed that behavioural alterations described in adults, as cognitive deficits, could represent sequelae of the acute phase of infection.^26^ Later, he suggested that the behavioural abnormalities could represent a slow, gradual, and progressive degeneration associated with the chronic infection.^37^ However, these studies lack appropriate socioeconomic traits- and age-matched non-infected control groups. Furthermore, some confounding aspects such as alcoholism, malnourishment, and co-infections were not considered.^24^ Independent of these not-considered parameters, the current biomedical model requires histopathological, visual, and mensurable data to support the clinical description of a chronic nervous form of CD. In general, well-conducted studies support the absence of neurological alterations in the chronic phase of *T. cruzi* infection. In the acute and chronic phases of *T. cruzi* infection of humans and experimental models, parasite forms are detected in the CNS parenchymal and perivascular cells,^27^ ^,^ ^38^ ^,^ ^39^ most of them expressing glial fibrillary acidic protein (GFAP), therefore, mostly astrocytes.^40^ Further, rare perivascular inflammatory infiltrates, low-grade oedema, sparce, and small inflammatory nodules in the white matter of the cortex, cerebellum, and nucleus dentatus, commonly associated with ruptured parasite-bearing cells were described in chronic CD patients^24^ ^,^ ^31^ ^,^ ^41^ and infected mice.^39^ Thus, consensually neuroinflammation in brain tissue, classically defined as the influx of blood-born inflammatory cells into perivascular cuffs and brain parenchyma,^6^ was discarded in chronic *T. cruzi* infection, ruling out the acceptance of the chronic nervous form of CD. However, one tip of the iceberg caught the eye. Histopathological study showed brain cortex atrophy, in the absence of neuroinflammation.^41^ Later, a computed tomography-based study with patients with *T. cruzi*-positive serology, compared with socioeconomic traits- and age-matched non-infected controls, revealed brain infarcts, focal oedema, and cerebral atrophy, independent of a structural cardiac disease related to chagasic cardiomyopathy.^42^ In this case, brain atrophy detected by histological studies and computed tomography may not be enough to fulfil the biomedical model requirements to settle a chronic nervous form of CD. However, the biological process unveiled, *i.e.*, brain atrophy, revealing the loss of neurons and glial cells, is crucial and may represent one tip of the iceberg supporting the progressive degeneration of the CNS associated with the chronic *T. cruzi* infection, as ingeniously proposed by Chagas.^37^

Behavioural and neurocognitive changes in chronic Chagas disease

A literature survey revealed well-performed studies showing that chronically *T. cruzi*-infected patients may exhibit: (i) a low quality of life, with cephalea, confusion, and depression;^43^ ^,^ ^44^ ^,^ ^45^ (ii) speech disturbance, depression and neurocognitive alterations and;^46^ and (iii) behavioural changes as anxiety and depression.^47^ ^,^ ^48^ There is no correlation between depression and Chagas’ heart disease, a clinical form of CD associated with chronic inflammation.^49^ Indeed, depressive symptoms have also been detected in patients with preserved cardiac function, and *T. cruzi*-positive serology seems to be a crucial determinant of depression.^50^ In quantitative electroencephalograms sleep dysfunction was detected, and these patients also show depression and memory impairment.^51^ Psychomotor alterations, attention and memory deficits have also been described in chronic CD patients.^45^ ^,^ ^52^ ^,^ ^53^

Association between *T. cruzi* infection and neurocognitive impairments has been shown using the mini-mental state examination score (MMES) to test elderly CD patients, which were not related to electrocardiographic alterations or the use of digoxin.^54^ Further, neurocognitive dysfunction in chronic CD patients has been supported by a deficiency in orientation, attention, non-verbal reasoning, information processing and learning.^44^ ^,^ ^51^ More importantly, memory impairment has been described in chronic CD patients as sequelae of acute meningoencephalitis^55^ or without apparent relation with the acute phase of *T. cruzi* infection.^56^ ^,^ ^57^

CD patients may present psychological traits concurring with behavioural changes.^58^ ^,^ ^59^ Psychological stress as being a carrier of an incurable illness, socioeconomic traits, and social discrimination, based on racism (most of the chagasic patients are mestizos) and low levels of education and income,^15^ ^,^ ^60^ have been raised as contributors to behavioural changes as anxiety and depression.^25^ Nevertheless, biological stressors may also trigger or aggravate behavioural and neurocognitive changes in CD patients, as previously proposed for non-infectious diseases neurodegenerative disorders such as Parkinson and Alzheimer disease.^2^ ^,^ ^61^ Among the biological stressors contributing to behavioural and neurocognitive changes are genetic-borne traits, a diet poor in nutrients essential to produce neurotransmitters, infection-induced damage in the CNS, and local and/or systemic oxidative stress and chronic inflammation.^2^ However, these features have been poorly explored in CD.

A chronic nervous form of CD has been ruled out based on the absence of neuroinflammation.^24^ ^,^ ^41^ However, the original hypothesis of Carlos Chagas of a slow, gradual, and progressive degeneration of the central nervous tissue^37^ and the findings of cortical atrophy with rare inflammatory nodules in the CNS parenchyma^41^ ^,^ ^42^ moved us to raise the idea that brain atrophy may represent the main anatomical substrate of cognitive impairment in CD patients. Further, these proposals led us to attempt to replicate some aspects of the behavioural and neurocognitive changes described in patients using preclinical models. Moreover, our main goal was to open new perspectives to explore the participation of infection of the CNS cells, neurochemical alterations, and/or systemic inflammatory profile in these biological processes in the scenario of chronic *T. cruzi* infection. These ideas have brought us here, although not by straight paths.

Behavioural and neurocognitive changes in preclinical models of Chagas disease: they insist on luring us

*“It seems that many things in life draw attention to the heart but are always best enjoyed when the brain joins the party*”, said a special someone.

About 20 years ago, we were interested in understanding the pathogenic mechanisms of chronic Chagas’ heart disease. Our paradigm was based on identifying biological and molecular mechanisms, to draw rational therapeutic targets aiming to improve prognosis.^19^ In Chagas’ heart disease, low-level cardiac inflammation is linked to low-grade parasite persistence, tissue injury, progressive fibrosis, electrical abnormalities, and dysfunction, potentially evolving to heart failure.^18^ ^,^ ^19^ The progression from IND to CARD form, and its severity (mild, moderate, severe), have been directly associated with the levels of the systemic inflammatory profile, revealed as increased serum levels of inflammatory (TNF, IL-6, IL-17, IFNγ, CCL2/MCP1) and regulatory/anti-inflammatory (IL-4, IL-10, TGFβ) cytokines, inflammatory mediators as nitric oxide, and reduced anti-oxidant enzymes as superoxide dismutase (SOD) and glutathione peroxidase (GPx), supporting inflammatory/anti-inflammatory and oxidant/anti-oxidant imbalance.^62^ ^,^ ^63^ This is a quite relevant trait. The long-lasting high levels of serum cytokines present in CD patients are recognised as a pivotal feature regarding behavioural and neurodegenerative diseases such as major depressive disorder, Parkinson and Alzheimer disease.^2^ ^,^ ^6^ ^,^ ^64^ More recently, the IL-6- and TNF-enriched cytokine storm detected in plasma has been proposed as a trigger of neurological and behavioural changes in Covid-19 patients.^64^ ^,^ ^65^

Thus, initially combining mouse lineages and *T. cruzi* strains we replicated relevant clinical traits of the cardiac disease, as electrical and structural heart changes, showing a direct relationship between the parasitaemia levels and the intensity of heart inflammation in the acute phase and the severity of heart disease in the chronic phase, as electrical and structural dysfunction, and fibrosis.^66^ ^,^ ^67^ ^,^ ^68^ Further, we described the association between the severity of heart alterations and the serum levels of cytokines and nitric oxide,^67^ replicating pivotal aspects of Chagas’ heart disease.^62^ ^,^ ^63^ Later, we showed a direct relationship between serum and cardiac tissue TNF levels and electrical abnormalities.^68^ ^,^ ^69^ ^,^ ^70^ These preclinical models were used to study aspects of CD pathogenesis, trials of immunoregulatory drugs,^68^ ^,^ ^69^ therapeutic vaccines,^71^ and combined multi-therapeutic strategy targeting parasite and inflammation-related alterations in chronic *T. cruzi* infection,^72^ ^,^ ^73^ opening opportunities to test new therapeutic schemes aiming to improve the prognosis of chronic Chagas cardiomyopathy.^19^ While performing these experiments we observed that chronically *T. cruzi*-infected mice were not interested in the objects used to enrich the cage as paper tubes and plastic igloo, compared with non-infected controls. More broadly, these findings led us to consider the presence of behavioural changes in preclinical models of chronic *T. cruzi* infection, opening new pathways to be explored.

Initially, we showed that C3H/He and C57BL/6 mice infected with low inocula of the Colombian *T. cruzi* strain, respectively, are susceptible and resistant to acute meningoencephalitis. This neuroinflammation is self-resolving in C3H/He. Thus, in chronically *T. cruzi*-infected C3H/He and C57BL/6 mice meningoencephalitis and neuroinflammation are absent. In an apparent silent way, rare amastigote-bearing GFAP^+^ cells are detected mostly surrounding blood vessels, as perivascular astrocytes, or scattered in the brain parenchyma.^32^ ^,^ ^39^ ^,^ ^40^ ^,^ ^74^ In the acute phase of the infection, endothelial cells of the CNS blood vessels are permissive to the adhesion of leukocytes, a process associated with oxidative stress.^75^ In the chronic *T. cruzi* infection, the peripheral blood cells are activated and express cell adhesion molecules and chemokine receptors and, therefore, potentially able to migrate and invade damaged tissues in humans^76^ ^,^ ^77^ and mice.^16^ ^,^ ^32^ However, rare mononuclear cells are restricted to specific areas of the incomplete blood-brain barrier of the CNS, such as choroid plexus, but they do not invade the brain parenchyma.^39^ ^,^ ^78^ In chronic infection, chemokines are not expressed in the encephalon and the endothelial cells of the CNS blood vessels are not permissive to cell adhesion.^32^ ^,^ ^74^ Altogether, these data may explain the absence of neuroinflammation in the chronic phase of CD.^24^ ^,^ ^41^

Crucially, we described in specific pathogen-free C57BL/6 mice chronically infected with the Colombian *T. cruzi* strain anxiety, depressive-like behaviour, and motor coordination disorder, independently of sickness behaviour (temperature changes, body weight loss) and neuromuscular disorder.^79^ ^,^ ^80^ It is an important finding as sickness behaviour and muscle weakness may confound the interpretation of behavioural tests.^81^ Acute and chronically infected rats present sleep and memory deficits.^82^ Recently, we described progressive memory loss in chronically *T. cruzi*-infected C57BL/6 mice.^78^ Notably, altogether these data support a contribution of biological stressors to trigger or aggravate behavioural changes and neurocognitive deficits in chronic *T. cruzi* infection.

Hence, using appropriate tests to access behavioural and cognitive performance in mice, we showed that chronically Colombian-infected C57BL/6 mice exhibit: (i) reduced motor coordination (decreased latency in the rotarod test); (ii) loss of innate compulsive behaviour (increased latency and reduced number of hidden balls in marble-burying test); (ii) anxiety (reduced number of entries and time spent in the open arms in elevated plus maze test and reduced number of central lines crossed in the open-field test); (iii) depressive-like behaviour (increased time of immobility in tail suspension test and forced swim test), and (iv) progressive memory impairment: object recognition memory loss (reduced discrimination index evaluated by the novel object recognition test), habituation memory deficit (reduced discrimination index in the open field test), partial loss of aversive memory (reduced latency in the shock evoked test), as we have shown in the recent years.^78^ ^,^ ^79^ ^,^ ^80^ Therefore, chronically *T. cruzi*-infected mice replicated pivotal aspects of behavioural and neurocognitive changes (anxiety, depression, memory loss) described in CD patients, as discussed above. Importantly, in chronically infected mice all these behavioural alterations occurred in the presence of encephalon atrophy and the absence of neuroinflammation,^32^ ^,^ ^78^ replicating other key features described in CD patients.^41^ ^,^ ^42^ Thus, our findings opened the opportunity to study the physiopathogenesis and the interconnectedness of these biological processes, further allowing the proposal of therapeutic strategies.

**Neurochemical alterations in depression: the case of chronically *T. cruzi* - infected mice**

A complex network of neurotransmitters takes part in physiological pathways controlling behavioural and neurocognitive performance. An appropriate diet with consumption of foods enriched in tryptophan is essential for serotonergic pathway with the production of 5-hydroxytryptophan (5-HTP) and serotonin (5-HT), whereas kynurenine pathway and imbalance of tryptophan metabolism towards kynurenine requires the indoleamine 2,3-dioxygenase enzyme (IDO) upregulation, associated with depressive behaviour.^83^ Crucially, the pro-inflammatory cytokines TNF and IFNγ enhance IDO-mediated tryptophan degradation and tryptophan catabolites (TRYCATs), with relevant neuropsychiatric implications since these catabolites may induce neurodegeneration and tryptophan is a serotonin precursor. Thus, IDO fluctuation in the CNS may affect serotonin availability and contribute to depression.^84^ More recent data questioned these points as depression is a more complex process than the originally thought imbalance of serotonin availability.^85^

In acutely *T. cruzi*-infected mice, increased IDO expression is detected in the spleen and muscles and associated with resistance to infection.^86^ In chronically *T. cruzi*-infected mice showing depressive-like behaviour, IDO upregulation was detected in the CNS. Further, the administration of the selective serotonin reuptake inhibitor (SSRI) fluoxetine abrogated the *T. cruzi*-triggered depressive-like behaviour.^79^ Interestingly, this antidepressant has anti-inflammatory activity, inhibiting NF-κB activation, producing a balanced immune response with decreased IFNγ and upregulated IL-10 expression,^87^ ^,^ ^88^ allowing the recovery of a less inflammatory scenario.

The detection of depressive behaviour in patients with chronic inflammatory disorders such as arthritis or hepatitis C-seropositive patients undergoing immunotherapy with IFNα has allowed the connection of immune response with an increase in cytokine levels and IDO-induced imbalance of tryptophan metabolism towards kynurenine pathway.^83^ ^,^ ^89^ Chronically *T. cruzi*-infected C57BL/6 mice show increased TNF levels in serum and elevated TNF expression systemically, including heart tissue, but not in the CNS.^67^ ^,^ ^74^ ^,^ ^79^ Initially, we challenged the participation of the TNF/TNFR1 pathway in depressive-like behaviour in the preclinical model of chronic CD. Importantly, administration of the anti-TNF neutralising monoclonal antibody infliximab^69^ or the TNFR1 modulator pentoxifylline^71^ ^,^ ^90^ reduced immobility in tail suspension and forced swim tests,^79^ supporting the participation of TNF/TNFR1 signalling pathway in depressive-like behaviour in this preclinical model of CD. Curiously, interventions at TNF/TNFR1 signalling using anti-TNF (infliximab) and pentoxifylline have been proposed to treat mood disorders such as depressive and bipolar disorders.^91^ Hopefully, it will open up possibilities for treating behavioural changes also described in patients with neglected diseases, as preclinical trials have already paved the way in the case of CD.

Stressor as the inflammatory cytokines IL-6, TNF and IFNγ have emerged as triggers of depressive behaviour.^84^ Pathogen-borne molecules such as lipopolysaccharide (LPS)^92^ and the HIV Tat protein^93^ induce IL-6 and TNF upregulation. Besides increases in the production of IFNγ and TNF, enhanced activation of IDO induced by Bacillus Calmette-Guerin (BCG) has been shown to be associated with depressive-like behaviour in mice,^94^ suggesting that this may be a pathway shared by *T. cruzi* infection. For sure, it is needed to study the molecular mechanisms underpinning the beneficial role of TNF/TNFR signalling abrogation on the depressive behaviour of chronically *T. cruzi*-infected mice. Nevertheless, these findings disclosed the possibility that in CD patients, besides psychological alterations, neurochemical pathway abnormalities could play a role in behavioural alterations. Thus, our data in preclinical models opened new therapeutic pathways to be explored to improve the quality of life of CD patients diagnosed with depression. For sure, access to diagnosis of behavioural changes is a bottleneck to be solved to attend patients afflicted by neglected diseases, such as CD.

**Intrinsic brain cytokine network supports *T. cruzi* parasite persistence in the glial cells**

Behavioural and neurocognitive alterations in CD patients and experimental models of *T. cruzi* infection occur in the absence of neuroinflammation, *i.e.*, in the lack of infiltration of the CNS by peripheral inflammatory cells,^41^ ^,^ ^78^ and apparently preserved parenchymal cells and neurons.^24^ ^,^ ^27^ Nevertheless, molecular pathways that may contribute to neurodegenerative signs as seen in taupathies,^95^ synucleinopathies,^96^ and other CNS damage paths await to be explored in *T. cruzi* infection. Also, it remains to be investigated the role of cellular and molecular mechanisms that dampen the immune response, such as induction of CD300f expression in glial cells,^97^ which could prevent neuroinflammation and allow parasite survival in the CNS. Indeed, a sharper analysis of the CNS of acute and chronically *T. cruzi*-infected mice revealed parasite persistence, mainly in GFAP^+^ cells subjacent to blood vessels.^40^ Anyhow, in the CNS most of the *T. cruzi*-carrying cells were situated near IFNγ^+^IBA-1^+^ cells. Of course, IFNγ may only represent a fingerprint associated with the release of other cytokines or effector molecules in these IFNγ^+^ microglial cells. Pre-treatment of primary astrocyte cell cultures with IFNγ increased parasite uptake and proliferation, regardless of nitric oxide production.^40^ Interestingly, IFNγ promotes HIV infection of the human astroglioma cell line U-87,^98^ inducing an antagonist of the β catenin pathway, in a STAT3-dependent manner, and, therefore, in this condition IFNγ downmodulates pathways related to virus replication control.^99^ In a situation of immunosuppression by HIV infection, the CNS is the main target tissue of the reactivation of *T. cruzi* infection.^29^ ^,^ ^35^ Curiously, *in vitro* experiments revealed that HIV and *T. cruzi* coexist in the same astrocyte cell, likely favouring reciprocal interactions proposed to be linked to an IL-6-promoted cytoprotective activity.^100^ Thus, the presence of IFNγ may promote the infection of astrocytes by both pathogens, a matter to be further mechanistically explored. The molecular routes facilitating *T. cruzi* uptake and growth in astrocytes by an IFNγ-enriched milieu remain to be clarified. The initial approach shows that contrasting with the NLPR3-dependent control of *T. cruzi* in microglial cells, this inflammasome is not activated in *T. cruzi*-infected murine astrocytes.^101^ This may be a decisive key as human cortex astrocytes do not express NLPR3,^102^ which may facilitate the survival of infectious agents in these cells. Critically, the inhibition of nitric oxide synthesis by L-NAME partially abrogated, while the anti-TNF monoclonal antibody completely blocked, IFNγ-induced *T. cruzi* uptake by astrocytes.^40^ These findings implicate IFNγ-stimulated astrocytes as sources of nitric oxide and TNF, which may take part in parasite persistence in the CNS in CD. Interestingly, the number of *T. cruzi*-bearing cells is reduced as the infection progresses from the acute to the chronic phase.^40^ Still, IFNγ (or any other simultaneously produced effector molecules) may play a dual role in the CNS, shutting down the high parasite burden in the acute infection, while fuelling low parasitism in the chronic phase of infection.

The pro-inflammatory cytokine TNF is involved in depressive-like behaviour in chronically *T. cruzi*-infected mice.^79^ Priming of mouse primary astrocyte cell cultures and human astroglioma cell line U-87 with TNF increased parasite/astrocyte interactions, multiplication of intracellular forms, and release of trypomastigote forms. Thus, TNF priming allowed the complete parasite cycle in astrocytes,^103^ mimicking the described in the CNS of CD patients.^27^ Further, *T. cruzi* infection of astrocytes creates a pro-inflammatory profile,^104^ particularly enriched in TNF and IL-6, a process amplified by priming with TNF and conserved in mouse and human astrocytes.^103^ Moreover, TNF treatment of astrocytes before infection upregulates TNFR1 expression, reinforcing the need for two signals to enhance TNFR1 expression. Crucially, pentoxifylline, a down-regulator of TNFR1 expression,^90^ abrogates the TNF-induced increase of susceptibility of astrocytes to parasite infection.^103^ Therefore, the TNF-enriched inflammatory milieu and the enhanced TNFR1 expression may favour TNF signalling in a self-sustained circuit. Lastly, cytokine-driven traits may perpetuate parasites in the CNS^40^ ^,^ ^103^ and promote behavioural alterations in the chronic phase of *T. cruzi* infection.^79^ It remains to be clarified whether pentoxifylline therapy plays a beneficial role in parasite control in the CNS, and behavioural and mnemonic changes in chronic *T. cruzi* infection. Astrocytes, major components of the blood-brain-barrier, which play a pivotal role in vascular permeability control, homeostasis, neuron protection, and local immune response,^102^ may be targets of viruses and parasites infection and promote a long-lasting invader persistence in the CNS. In this context, our data suggest that cytokine-driven microglial cells/astrocytes interaction may favour *T. cruzi* persistence in brain tissue, mainly alberged in astrocytes, and play a pivotal role in the CNS commitment in chronic CD (Fig. 2A).

Systemic inflammatory profile with enhanced serum concentrations of cytokines and inflammatory mediators (such as IFNγ, IL-6 TNF, TGFβ and nitric oxide) are directly associated with the severity of chronic chagasic cardiopathy, while the levels of antioxidant enzymes such as SOD and GPx are inversely correlated with disease.^62^ ^,^ ^63^ ^,^ ^77^ Chronically *T. cruzi*-infected mice also show a systemic inflammatory profile, with increased serum levels of proinflammatory (mainly IL-6, IFNγ, and TNF) and regulatory (IL-10) cytokines (Fig. 2B). Thus, in the presence of *T. cruzi* infection, these inflammatory mediators may access the CNS via vagus nerve (neural pathway), regions of disrupted or incomplete blood-brain-barrier, such as choroid plexus (humoral pathway),^61^ creating an inflammatory milieu, which may activate astrocytes, microglial cells, and neurons. Therefore, in chronic CD the out/in input of inflammatory mediators in the CNS may activate glial cells and/or neurons creating a self-sustaining cytokine-driven inflammatory milieu putatively implicated in parasite persistence, inducing neurochemical changes (as IDO upregulation, oxidative stress, neurotransmitters), and trigger behavioural and neurocognitive abnormalities (Fig. 2A). This is a matter to be further mechanistically explored not only in CD but also in other infectious diseases featured by enhanced systemic cytokine levels (for some called “cytokine storm”) as TNF and IL-6, as COVID-19,^65^ ^,^ ^105^ septic shock,^106^ ^,^ ^107^ and leprosy.^108^ ^,^ ^109^ In summary, this more broadly idea is exposed in Fig. 3, proposing that in the acute phase of infection (weeks or months) IL-6 and TNF may take part in microbial control, but also contribute to behavioural and/or neurocognitive alterations, which may be total or partially recovered; however, long-lasting (years) systemic inflammation enriched in these cytokines may lead to long-term behavioural and neurocognitive changes.

**Fig. 2:**
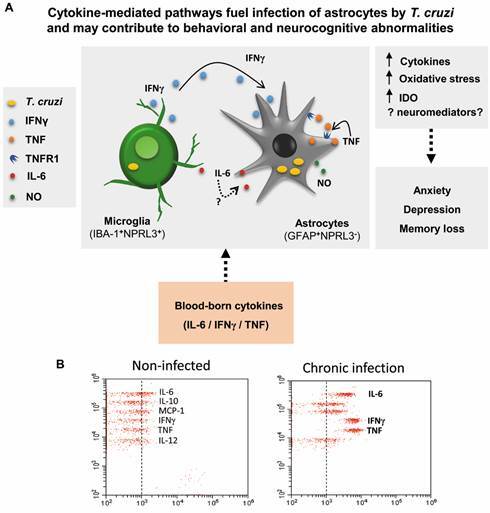
theoretical proposal of cytokine-mediated pathways fueling infection of glial cells by *Trypanosoma cruzi* and contributing to behavioral and neurocognitive abnormalities. (A) Schematic proposal. In chronic Chagas disease (CD) the out/in input of inflammatory mediators (such as the blood-borne cytokines IL-6, IFNγ and TNF) in the central nervous system (CNS) may activate glial cells (microglia - IBA-1^+^NPRL3^+^- and/or astrocytes - GFAP^+^NPRL3^-^),^40^ ^,^ ^101^ ^,^ ^103^ creating a self-sustaining cytokine-driven inflammatory milieu putatively implicated in parasite persistence^40^ ^,^ ^103^ and inducing neurochemical alterations, such as indoleamine 2,3-dioxygenase enzyme (IDO) upregulation,^79^ oxidative stress^78^ and neuromediator changes (to be identified). These inflammatory alterations may trigger behavioral and neurocognitive abnormalities (anxiety, depression and memory loss).^78^ ^,^ ^79^ ^,^ ^80^ Cytokines and inflammatory mediators such as cytokines (IL-6, IFNγ and TNF) and nitric oxide (NO) may take part in parasite persistence in the central nervous system, fueling parasite growth.^40^ ^,^ ^103^ TNF/TNFR1 signaling may be crucial for parasite infection and persistence in astrocytes.^103^ (B) Representative dot plots of inflammatory cytokines in the serum of non-infected, and chronically Colombian-infected C57BL/6 mice showing clinical signs of behavioral and neurocognitive alterations,^78^ ^,^ ^79^ detected as previously described.^137^

**Fig. 3:**
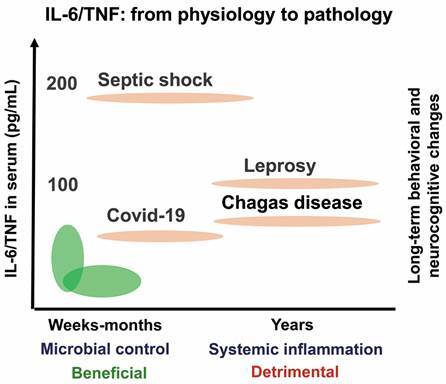
theoretical proposal of IL-6/TNF participation in physiology and pathology in several infectious diseases. Elevated levels of IL-6 and TNF in the acute phase of infection (weeks or months) may take part in microbial control (beneficial), but also contribute to behavioral and/or neurocognitive alterations, which may be total or partially recovered. However, long-lasting (years) IL-6- and TNF-enriched systemic inflammation may access the central nervous tissue and lead to long-term behavioral and neurocognitive changes (detrimental). Elevated levels of cytokines in serum may be a common trait in several infectious diseases such as Covid-19,^65^ ^,^ ^105^ septic shock,^106^ ^,^ ^107^ leprosy,^108^ ^,^ ^109^ and Chagas disease (CD).^62^ ^,^ ^63^ ^,^ ^77^

**Parasite participation in neurobehavioural and mnemonic alterations in the chronic phase of *T. cruzi* infection**

The pivotal participation of the *T. cruzi* parasite in neurobehavioural alterations has initially been proposed by Chagas and Vianna.^27^ ^,^ ^37^ Later, the recognition of *T. cruzi* infection as an opportunistic infection in HIV^+^ patients,^110^ opened a field to be explored. Hundreds of cases of HIV/*T. cruzi* infection have been described, as showing neurobehavioural changes, such as mental confusion and convulsion.^33^ The central participation of *T. cruzi* in these processes has been confirmed when benznidazole therapy was timely administered, with effective parasite control and brain lesion resolution.^29^ ^,^ ^35^ ^,^ ^111^ These data reinforce the accessibility of the CNS through benznidazole therapy.^36^

The impact of parasite diversity on the CNS injury and neurobehavioural alterations has been explored. In a case report of lethal CD reactivation, polyclonal populations with diversity of typing unit (DTU) were detected in blood (DTU I and IId/e), however, only DTU-I was detected in the CNS.^112^ It remains to be clarified whether it reflects experimental limitations or a biological trait, as selective tropism of DTU-I for the CNS and/or parasite control in this tissue. Anyhow, congruently with this finding depressive-like behaviour was triggered by the DTU-I Colombian strain, but not by the DTU-II Y strain, although both parasite populations colonise the CNS of mice.^79^ A question to be further explored considering the genetic and biological diversity of *T. cruzi*.

Other crucial points to be better explored are the routes and mechanisms of colonisation of the CNS by *T. cruzi* parasite: (i) blood-born parasites may infect endothelial cells of the CNS blood vessels, get access to perivascular GFAP^+^ cells and parenchymal glial cells;^40^ (ii) areas of incomplete blood-brain barrier of the CNS, such as choroid plexus, as trypomastigote forms may be detected in cerebrospinal fluid;^29^ (iii) Trojan horse as macrophages and other cells carrying amastigote forms may adhere to activated blood vessels and enter the brain tissue;^113^ (iv) palate or olfactory neuroepithelium in oral or nasal infection;^114^ (v) vagus nerve as in case of gastrointestinal commitment. Indeed, all these routes have been described for other microbials, including protozoans, to invade the CNS.^115^ ^,^ ^116^ However, one should keep in mind that more than the access of the CNS by the parasite itself, systemically released extracellular vesicles of *T. cruzi* forms and/or infected cells may cross the blood-brain-barrier, interact with glial cells and neurons, stimulate the production of cytokines and other inflammatory mediators, which may trigger tissue, cellular and molecular alterations related to behavioural and neurocognitive changes.^117^ These are matters to be further explored, particularly aiming to understand parasite/host interactions and to reduce the access of *T. cruzi* parasite forms and products into the CNS, via vaccines (using proper routes, such as oral and intranasal) and etiological therapies.

Cognitive dysfunction has been associated with chronic CD.^44^ ^,^ ^51^ ^,^ ^54^ ^-^ ^57^ ^,^ ^118^ In recent work, we showed that DTU-I-*T. cruzi*-infected C57BL/6 mice show progressive memory impairment. Firstly, all chronically infected mice lost memory of object recognition, followed by habituation memory impairment and progressive decline of aversive memory, compared with age-matched controls.^78^ Crucially, administration of a low dose of benznidazole for short period (30 days) to chronically infected mice showing a deficit of object recognition memory reversed this mnemonic alteration and hampered the progression of habituation and aversive memory loss. Neuroinflammation was absent in chronically infected mice with memory deficits, and benznidazole therapy did not affect the CNS architecture. As expected, benznidazole treatment reduced parasite load in the hippocampus and cerebral cortex.^78^ In degenerative diseases, oxidative stress, revealed by enhanced lipid peroxidation in the cerebral cortex, has been liked to memory dysfunction and neurodegeneration.^119^ Lipid peroxidation was increased in extracts of hippocampus and cortex tissue of chronically *T. cruzi*-infected mice. Remarkably, the trypanocidal therapy reduced levels of lipid peroxidation in the cerebral cortex.^78^ We cannot exclude, however, the possibility that besides the trypanocidal activity, benznidazole is playing an indirect immunomodulatory effect in the CNS. In a murine model of sepsis, benznidazole therapy reduced lipoperoxidation and the expression of cytokines and cell membrane TLR4 in hepatic tissue, in a way involving protein kinase and Nfr2-induced antioxidant mechanism.^120^ Therefore, direct or indirect parasite-induced immune dysregulation may contribute to chronic behavioural and neurocognitive alterations in *T. cruzi* infection. Thus, benznidazole therapy may decrease systemic parasitism and parasitaemia, reducing the invasion and parasite load in the CNS and, consequently, glial cell activation and cytokine-driven parasite growth in these cells,^40^ ^,^ ^78^ ^,^ ^103^ improving behavioural and neurocognitive alterations.^78^ ^,^ ^79^

The chronic CD is a risk factor for stroke, regardless of the severity of cardiomyopathy. Indeed, stroke is an important neurobehavioural change in CD, leading to chronic disabilities.^121^ ^,^ ^122^ ^,^ ^123^ Although most strokes are cardioembolic, related to apical aneurysm, mural thrombus, and atrial fibrillation, small vessel diseases and atherosclerosis may also concur to stroke in CD patients. Thus, endothelial dysfunction, and prothrombotic and inflammatory disorders may represent non-embolic risk factors for stroke in CD. Even though the pathophysiological mechanisms are not completely understood, clinical approaches for the prevention of stroke in CD are demanded.^123^ Interestingly, in a long-term follow-up benznidazole therapy was associated with a decreased incidence of CD progression from the IND to the CARD form, and with a reduced risk of cardiovascular events, including stroke.^124^ Considering the putative effect of benznidazole on biological mechanisms contributing to stroke, our studies in chronically *T. cruzi*-infected mice showed that benznidazole therapy abrogated atrial fibrillation and reduced the expression of chemokines and cytokines (such as CCL3, TNF, and IFNγ) in the cardiac and the systemic inflammatory profile, as decreased the TNF and nitric oxide serum levels.^19^ ^,^ ^72^ ^,^ ^73^

Although the molecular mechanisms sustaining the beneficial effects of benznidazole systemically and, particularly, in the CNS are not known, our data provide a new perspective to ameliorate the neurobehavioural, depressive, and neurocognitive disorders and improve the quality of life of chronic CD patients. Indeed, therapies aiming to control the *T. cruzi* parasite are a need to add advantage to immune-mediated parasite control in the chronic phase of CD and impact the onset and progression of clinical signs. Sadly, most of the *T. cruzi*-infected individuals miss diagnosis and a more effective time window to offer etiological treatments. Benznidazole is considered highly effective in the acute phase (60-80%), and drug efficacy is reduced in the chronic phase of the infection (20-60%), possibly due to commitment of the immune response in aged patients and/or differences in susceptibility to drugs among the different *T. cruzi* lineages.^10^ ^,^ ^21^ Several clinical trials (TRAENA, CHAGASAZOL, TESEO, BENDITA) are being conducted to reduce the dose and/or time of administration of benznidazole, aiming at reducing side effects and increasing adhesion to etiological therapy.^21^ Presently, new drugs targeting parasite-restrict biological pathways and metabolic vias are under development.^21^ ^,^ ^125^ It is hoped that new medicaments will be soon available and, as here announced by benznidazole therapy, impact neurobehavioural, depressive, and neurocognitive disorders.

Final remarks

Age-related physiological deterioration may exacerbate multiple pathogenic processes, priming the brain for neurodegeneration.^119^ Here, we propose the hypothesis that *T. cruzi* infection, inducing chronic inflammation, neuro-hormonal alterations, and oxidative stress may trigger or accelerate the process of aging in the CNS, opening a sequential installation of behavioural and neurodegenerative processes. Naturally, it remains an avenue to be paved. Further, basic questions still await to be addressed in CD patients, such as the contribution of gender-linked factors (chromosomes, genes, hormones) shown to contribute to behavioural and neurodegenerative alterations in other illnesses, particular in aging females.^126^ As an iceberg tip, a higher prevalence of depression has been shown in women compared to men with CD,^50^ calls attention to this topic.

The study of the cellular and molecular basis of the pathogenesis of the neurobehavioural (stroke), behavioural (anxiety, depression), and neurocognitive (learning and memory) changes in chronic CD patients and experimental models may add important information to rational and target-driven therapeutic strategies. Indeed, many questions regarding *T. cruzi*/glial cells/neurons crosstalk, using *in vivo* and *in vitro* models, and the neurocognitive changes in chronic CD patients and experimental models have been neglected. Among these pathways, one may list the misfold, degradation, and accumulation of proteins associated with neurodegenerative diseases, such as Tau, α-synuclein, TDP-43, and amyloid-β,^127^ ^,^ ^128^ as well as age-related, neuroimmune and inflammatory changes.^1^ ^,^ ^2^ Classically defined neuroinflammation, with influx of blood-born inflammatory cells in perivascular cuffs and parenchyma,^6^ is not detected in the CNS of chronic CD patients and experimental models.^24^ ^,^ ^32^ ^,^ ^39^ ^,^ ^41^ ^,^ ^78^ However, parasite persistence mainly in GFAP^+^ cells surrounded by IFNγ^+^IBA-1^+^ glial cells in chronically infected mice^40^ supports that specialised immune response is taking place in the CNS, an issue to be further explored. In this context, the participation of meningeal immunity^129^ in the fate of glial and neuronal cells as well as in behavioural and neurocognitive changes in *T. cruzi* infection remains an intriguing question for future research.

Another point to be further explored is the gut-brain imbalance contributing to dysbiosis and a putative relation between the gastrointestinal form of CD^130^ and behavioural and neurocognitive changes. Also, the microbiota-gut-brain axis gained attention due the role played by gut-microbiota in a myriad of biological circuits crucial for behavioural and neurocognition processes,^131^ including the regulation of tryptophan bioavailability and its conversion to neuroactive metabolites such as serotonin, melatonin, kynurenine and quinolinic acid,^131^ ^,^ ^132^ a matter to be explored in *T. cruzi* infection. In CD, diverse factors may concur to deterioration of the CNS, neurobehavioural changes (such as stroke)^123^ and trigger behavioural and neurocognitive alterations. It is also conceivable to consider that in CD patients CNS impairment may have a neuro-hormonal basis involving the renin-angiotensin-aldosterone system,^133^ ^,^ ^134^ since systemic and brain renin-angiotensin system may take part in aging and neurodegenerative disorders, through oxidative stress and vascular dysfunction.^135^

Regarding therapeutic approaches, besides the discussed role of etiological therapy,^78^ non-pharmacological strategies as physical activities, proposed as a cardioprotective strategy in CARD and IND conditions^136^ ^,^ ^137^ could also contribute to improving mental health of chronic CD patients. Physical exercises are suggested to improve depression^138^ and slow the progression of neurodegenerative processes,^139^ probably dampening common deleterious pathways as immune-neuroendocrine alterations (neurotransmitters and cytokines), oxidative stress, and mitochondrial dysfunction. Altogether, we have brought some perspectives to the discussion, hoping that more than academic achievement, the recognition of a chronic nervous form of CD, particularly involving neurobehavioural, behavioural, and neurocognitive changes, may attract young researchers to embrace these challenges in a neglected disease, affecting equally neglected persons.
